# Transmission risk beyond the village: entomological and human factors contributing to residual malaria transmission in an area approaching malaria elimination on the Thailand–Myanmar border

**DOI:** 10.1186/s12936-019-2852-5

**Published:** 2019-07-01

**Authors:** Hannah M. Edwards, Patchara Sriwichai, Kirakorn Kirabittir, Jetsumon Prachumsri, Irwin F. Chavez, Jeffrey Hii

**Affiliations:** 1 Department of Disease Epidemiology, Imperial College London, London, UK; 2 Malaria Consortium Asia, Faculty of Tropical Medicine, Mahidol University, 420/6 Rajvithi Road, Bangkok, 10400 Thailand; 3 Department of Entomology, Faculty of Tropical Medicine, Mahidol University, 420/6 Rajvithi Road, Bangkok, 10400 Thailand; 4 Mahidol Vivax Research Unit, Faculty of Tropical Medicine, Mahidol University, 420/6 Rajvithi Road, Bangkok, 10400 Thailand; 5 Department of Tropical Hygiene, Faculty of Tropical Medicine, Mahidol University, 420/6 Rajvithi Road, Bangkok, 10400 Thailand

**Keywords:** Malaria, RMT, Vector control, Vector behaviour, Forest malaria

## Abstract

**Background:**

A mixed methods study was conducted to look at the magnitude of residual malaria transmission (RMT) and factors contributing to low (< 1% prevalence), but sustained transmission in rural communities on the Thai–Myanmar border.

**Methods:**

A cross-sectional behaviour and net survey, observational surveys and entomological collections in both villages and forested farm huts frequented by community members for subsistence farming practices were conducted.

**Results:**

Community members frequently stayed overnight at subsistence farm huts or in the forest. Entomological collections showed higher biting rates of primary vectors in forested farm hut sites and in a more forested village setting compared to a village with clustered housing and better infrastructure. Despite high levels of outdoor biting, biting exposure occurred predominantly indoors, particularly for non-users of long-lasting insecticidal nets (LLINs). Risk of biting exposure was exacerbated by sub-optimal coverage of LLINs, particularly in subsistence farm huts and in the forest. Furthermore, early waking hours when people had left the safety of their nets coincided with peaks in biting in later morning hours.

**Conclusions:**

Entomological and epidemiological findings suggest drivers and modulators of sustained infection prevalence in the area to be: higher mosquito abundance in forested areas where LLINs were used less frequently or could not be used; late sleeping and waking times coinciding with peak biting hours; feeding preferences of *Anopheles* taking them away from contact with LLIN and indoor residual spraying (IRS), e.g. exophagy and zoophagy; non-use of LLIN and use of damaged/torn LLIN; high population movement across the border and into forested areas thereby increasing risk of exposure, decreasing use of protection and limiting access to healthcare; and, *Plasmodium vivax* predominance resulting in relapse(s) of previous infection. The findings highlight gaps in current intervention coverage beyond the village setting.

**Electronic supplementary material:**

The online version of this article (10.1186/s12936-019-2852-5) contains supplementary material, which is available to authorized users.

## Background

Malaria vector control in the Greater Mekong Sub-region (GMS) relies almost exclusively on long-lasting insecticidal nets (LLINs). LLINs reduce malaria parasite transmission mainly by killing, repelling or disabling mosquitoes that come into contact with their insecticidal netting as they attempt to feed upon humans sleeping under nets [1]. Although the evidence-base supporting the use of LLINs in the GMS is not as extensive as that in Africa, local randomized control trials have found significant improvements in malaria outcomes, particularly against the major vectors, *Anopheles dirus* sensu lato (s.l.) and, *Anopheles minimus* s.l. [2, 3]. For example, a study in Cambodia showed a 37% reduction in the prevalence of *Plasmodium falciparum* in children in villages provided with insecticide-treated nets (ITNs) compared to children in villages without bed nets [4]. On the Thailand–Myanmar border, children aged 4–15 years (n = 350) who were given ITNs exhibited 42% fewer symptomatic episodes, however, parasite prevalence rates were similar in treated and untreated bed net groups [5]. This lack of consistent protection by ITNs against malaria in children living in malaria endemic villages was due to strong preference for outdoor biting by secondary vector species (*Anopheles epiroticus* s.l., *Anopheles subpictus* s.l., *Anopheles maculatus* s.l., *Anopheles aconitus* s.l. and *Anopheles vagus* s.l.). Given the occurrence of 52 genetic forms in the GMS, of which about 39 forms remain unnamed and their exact species (sensu stricto) are indeterminate [6], a sensible approach is to delete sensu lato (s.l.) to mean any or all members of the species complex from this point onwards. Mosquito biting behaviour is thought to contribute to the persistence of malaria transmission in some areas where there is reportedly high coverage of LLIN. The purported limited effectiveness of mosquito nets in these settings may result from the heterogeneity of vector transmission ecologies across the region and overlap of vector feeding behaviour and human activity that in some circumstances increase contact and undermine the effectiveness of the control measure(s). Effectiveness of ITNs can be determined by the behaviour of key vector species in an area if those behaviours mean they avoid vector control measures, as is the case in Western Myanmar [7]. In the GMS, the early and outdoor biting behaviour of the primary vectors (*An. dirus* and *An. minimus*) including the sibling species of *Anopheles baimaii, An. maculatus* s.s., and *Anopheles sawadwongporni* varies both geographically and seasonally [6, 8]. *Anopheles dirus* has also been shown to be a highly efficient vector of artemisinin-resistant *Plasmodium* parasites, representing a significant challenge to the elimination of malaria in Southeast Asia and the prevention of spread of multi-drug resistance [9, 10].

On the human side, many of the key population groups with the highest burden of malaria exhibit behaviour or rely on occupations that take them away from the protection of ITNs at peak biting times, particularly communities that practice subsistence farming and/or stay overnight in forest farms where the housing is often completely or partly open [6, 11–13]. Consequently, the malaria vectors that preferentially bite outdoors will freely enter these open dwellings and complicate the indoor/outdoor biting distinction. High mobility, forest and farming practices of the high-risk groups in many of the remaining transmission areas mean that individuals may not only be at risk of transmission in their villages, where LLINs are targeted, but also in other ecological sites that are prime habitats for the principle malaria vectors, such as the farm, forest rest sites, waypoints and sites used for deep forest economic activities [6, 14]. The transmission that persists even after achieving universal coverage of effective LLINs and/or maximal coverage of indoor residual spraying (IRS) with insecticides containing active ingredients to which the local vector populations are fully susceptible, has been termed ‘Residual Malaria Transmission’ (RMT) [14]. This is analogous to the definition used by Killeen et al. [15]. As a result of the vector and human behaviours described, the fraction of transmission in the GMS that may be described as residual is probably higher than in many parts of Africa [14].

Thailand has aimed to complete countrywide elimination of malaria and prevention of re-establishment in malaria-free areas by 2024 [16], but this is hindered in many districts, particularly those in border areas, by the continuing presence of malaria infections in migrant workers or ethnic minority groups with ‘high risk’ occupations [17, 18]. To investigate the magnitude of RMT and its contributing factors, entomological and social-behavioural methods were applied across different ecological sites frequented by members of three neighbouring and high-risk villages in Thailand.

## Methods

### Study area

Communities were selected if they met the following inclusion criteria: a sustained level of malaria incidence despite apparent universal coverage of ITNs/LLINs reported by National Malaria Control Programme (NMCP)/local distribution data; practicing subsistence farming/slash and burn agriculture and travel into the forest; and, accessible to the survey teams.

The neighbouring villages of Suan Oi, Komonae and Pha Man in Tha Song Yang district situated along the Thai–Myanmar border in Tak province were selected, with populations of 531 (Suan Oi) and 301 (Komonae and Pha Man, administratively considered as two forested hamlets of the same village by the local government and NMCP) individuals in 2016 (government administrative figures, pers. commun.). The villages included mostly Myanmar migrants of Karen ethnicity. During the study period (June–November 2016), average night time temperature measured by a HOBO weather data logger during mosquito collections (July–November 2016) was 25.9 °C (ranging from 23.3 °C during November collections to 26.9 °C during September collections) and average relative humidity was 91.1% (ranging from 87.1% in July to 96.0% in August). Total monthly rainfall between June and November was 170.1 mm, ranging from 321 mm in October to 110 mm in November (government meteorological department figures, pers. commun.).

**Fig. 1:**
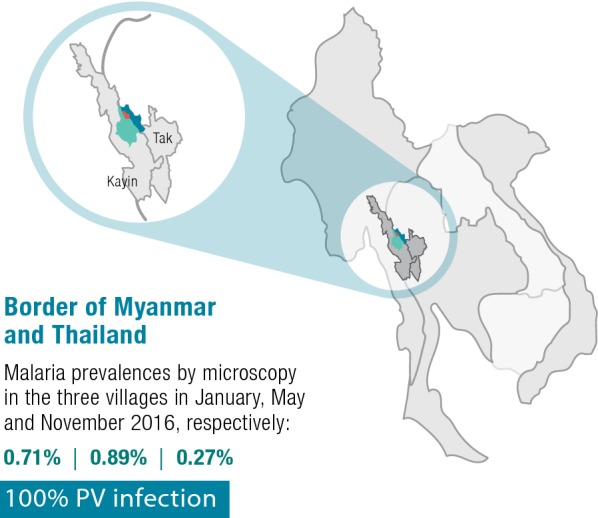
Location and malaria prevalence level of the study site within the Greater Mekong Subregion

Malaria incidence occurs all year round and in 2015 was reported to be 278 per 1000 population in Suan Oi and 148 per 1000 population in Komonae and Pha Man combined [figures from local vector borne disease control (VBDC) of the Thailand NMCP, pers. commun.]. As part of a concurrent longitudinal study in the area, prevalence by microscopy in the three villages was found to be 0.71%, 0.89% and 0.27% in January, May and November 2016, respectively (Fig. 1); all infections were caused by *Plasmodium vivax*.

**Fig. 2:**
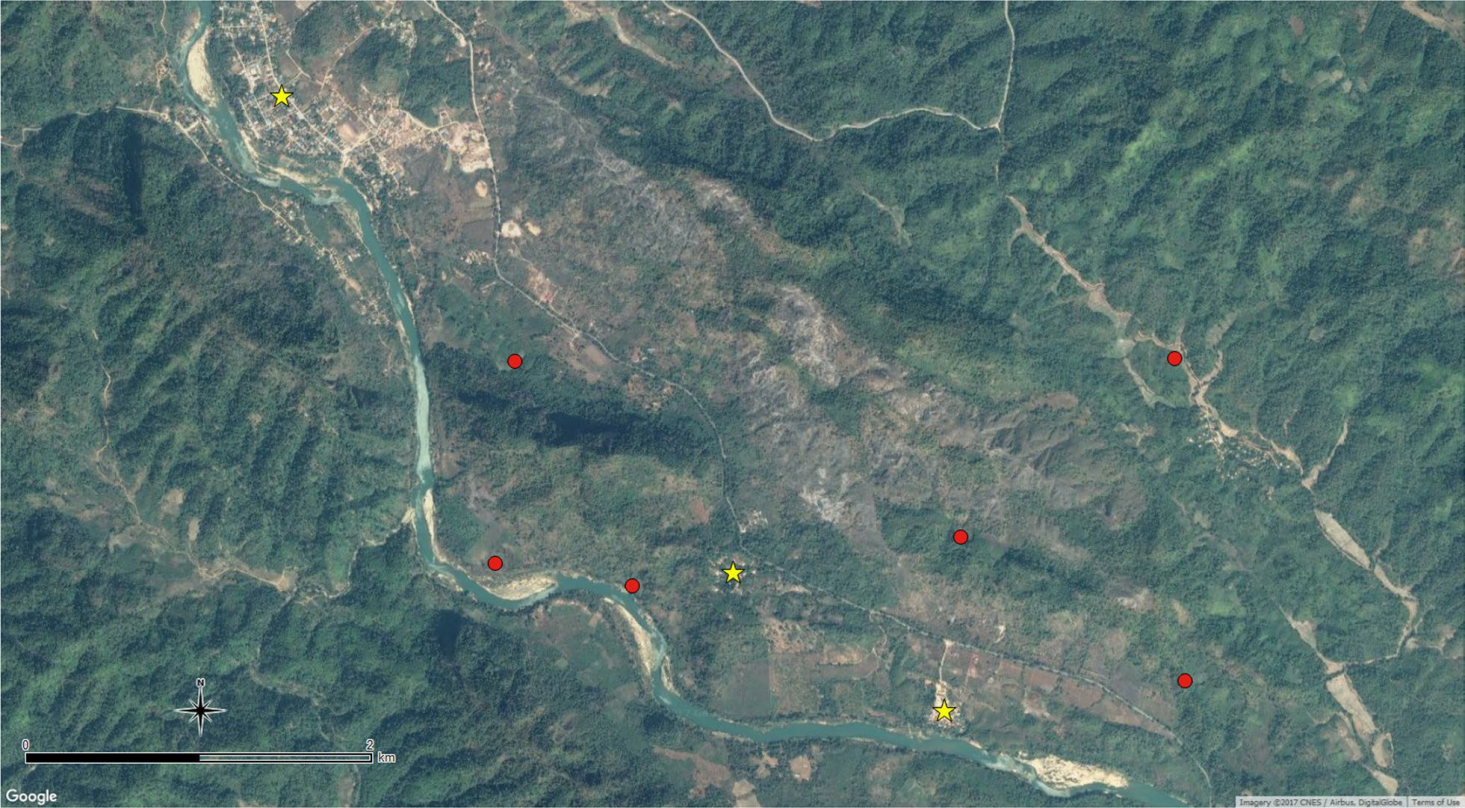
Map of entomological collection sites across villages and forested farm huts. Map shows study villages (yellow stars) and farm hut entomology collection sites (red circles). The river marks the boundary between Thailand and Myanmar

Suan Oi village is relatively urban and wealthy with clustered houses, relatively good infrastructure, health services and electricity; Komonae is situated along the river surrounded by open fields while Pha Man is situated on forested hilly ground slightly inland from the river (Fig. 2). Most houses are near a stream 1–10 m wide and some swamps which are likely mosquito breeding habitats, since mosquito larvae have been observed [19]. All three villages were included in the cross-sectional epidemiological survey but, due to resource constraints, only Suan Oi and Pha Man were selected for monthly village entomological collections to investigate a mix of ecologies. Primary vector species of interest in the area are *An. minimus* and *An. maculatus* [19]. *Anopheles dirus* had not been captured in this study area before but is still considered a primary vector species in Thailand, although its abundance may have declined due to its high vulnerability to IRS [20–22] and unfavourable ecological conditions for breeding in deforested areas [23, 24].

At the time of the study, the Thailand NMCP’s vector control policy recommended that IRS be conducted twice a year in perennial transmission areas (including the study site), and annually in periodic transmission areas. Permethrin LLINs were distributed free of charge and thermal fogging is applied during malaria outbreaks once a week for four consecutive weeks.

### Behavioural and net coverage survey

#### Data collection

A cross-sectional survey of adults was conducted in the target villages in September 2016. All adults aged 18 years and over were approached for participation in the survey and voluntary consent was obtained. Questions were asked about their demographic background, utilization of nets, farm and forest going habits. Heads of households were also asked about the size of their households, ownership of nets and the habits of any children aged less than 18 years that lived in the households. Type of nets were as reported by individual participants and were not verified in the field.

#### Data analysis

Data was analysed using Stata version 14 [25] to give proportions and confidence intervals (CIs) for key indicators related to household net coverage, utilization of nets the previous night, frequency of staying in the farm hut or forest, use of nets in the farm hut or forest, and sleeping hours. Population access to an ITN was calculated as previously recommended [26]. Firstly, the number of ITN in the household was multiplied by a factor of 2.0 to get the number of “potential ITN users”. To adjust for households with more than one net per two people, the potential ITN users was set to the de-facto population in that household. Then, the potential ITN users was divided by the number of household members as reported by the household head to determine the overall sample mean access.

### Entomological survey

#### Sampling

Mosquito collections were conducted in the villages and farm hut sites as shown in Table 1 and Fig. 2. Indoor (IHLC) and outdoor human landing catches (OHLC) were carried out for two consecutive nights per month from June to November in Suan Oi and Pha Man. Cow-bait collections were carried out concurrently for two consecutive nights per month from June to November in Suan Oi, and from July to November in Pha Man (no June collection was completed due to logistical constraints).

**Table 1:** Entomological collection nights conducted in each site and with each collection method

Site	Catch methods	Collection nights
Suan Oi village	IHLC; OHLC; cow-bait	Each method: Jun–Nov, two consecutive nights per month (12 person/cow-bait-nights)
Pha Man village	IHLC; OHLC; cow-bait	IHLC/OHLC: Jun–Nov, two consecutive nights per month (12 person-nights) Cow-bait: Jul–Nov, two consecutive nights per month (10 cow-bait-nights)
Farm huts (six sites in total: F1–F6)	Semi-indoor HLC	F1, F2, F3—three consecutive nights in Aug and Oct (6 person-nights each site) F4, F5—three consecutive nights in Oct and Nov (6 person-nights each site) F6—three consecutive nights in Oct (3 person-nights)
Total		103 collection nights (incl. 22 cow-bait nights and 81 person-nights)

*IHLC* indoor human landing catch, *OHLC* outdoor human landing catch

HLC at farm hut sites were conducted during August–November following selection of sites identified during a concurrent GPS tracking study which identified common sleeping and resting places used during time away from the village home. Six separate farm hut sites were selected from among the locations where participants spent nights away from the village that were also accessible for the survey team on the Thailand side of the border (Fig. 2).

Collections were conducted by trained village volunteers at each selected farm site for three consecutive nights at either one or two different time points (Table 1). Collectors wore a GPS tracker (brand: i-gotU GT-120 GPS data logger) to pinpoint the collection location and completed a short interview upon returning from the farm huts to confirm nature of the collection site. Collection sites were mapped visually using QGIS 2.18.14 [27].

In line with health and safety procedures, all volunteers were tested for malaria infection by microscopy 7 days before and after participating in mosquito collections, as well as monitored for malaria symptoms, and were excluded from participation if any positive symptoms were detected, as well as taken to the local health centre for appropriate treatment.

#### Collection method

For each HLC site two-person teams collected mosquitoes from 18:00–00:00 (person one) and 00:00–06:00 (person two). Each hour included 45 min collection and 15 min break to prevent fatigue. One person sat with their lower legs exposed and, using an aspirator tube and torch, collected any mosquitoes landing on their legs and transferred them into paper cups covered with netting and labelled by date, location and hour of collection. The cow-bait catch was conducted using a single cow in a tent-trap. Mosquitoes resting on the inside of the tent were collected using aspirators every 2 h from 18:00 to 06:00. At the end of each collection period, mosquitoes were transported back to the laboratory for processing.

In the farm huts, collectors remained there for three consecutive days and stored samples with silica gel before returning them to the field team after the third day. Since a high volume of mosquitoes were caught in the early evening during the first three farm hut collections in August, collection times in the farm huts were then extended to 17:00–07:00.

#### Identification of *Anopheles* species and infective status

*Anopheles* specimens were processed next morning in the laboratory according to time, study site and method of collection. All mosquitoes were morphologically identified using taxonomic keys developed by Rattanarithkul et al. [28]. The identified *Anopheles* mosquitoes were kept in silica gel contained in a sterile 1.5 mL microfuge tube and transferred from the field site in dry ice and stored at − 20 °C until determination of *Plasmodium* infection using sporozoite ELISA. Collected *Anopheles* mosquitoes were tested for malaria parasite sporozoites using the modified two-site sandwich ELISA described by Wirtz et al. [29, 30].

#### Entomological data analysis

Analysis was conducted for each site and method of collection separately to include abundance of anopheles, nightly and hourly biting rates and infection status was used to calculate Entomological Inoculation Rate (EIR, measured as the number of infected bites received per person per week). Since extended collection hours in the farm huts were conducted for 24 out of 33 collection nights, appropriate adjustments were made to mosquito numbers caught in the extended time to make hourly and nightly biting rates comparable. Rate of exophagy was calculated as HBR_O_/(HBR_O_ + HBR_I_), where HBR_O_ and HBR_I_ are the human outdoor and indoor biting rate, respectively. Rate of zoophagy was calculated as, CBR/(CBR + HBR_O_) where CBR is the cattle biting rate and outdoor human biting rate was used to make ecological sites comparable.

Counts of primary vector species—*An. dirus, An. minimus.* and *An. maculatus* were summarized as proportions. A multivariable Poisson regression model with negative binomial error and log link function was used to assess if the collection methods (IHLC, OHLC) and collection sites (indoor, outdoor, and farm huts) were independently associated with anopheline abundance. A series of likelihood ratio tests were done to determine the most suitable models. Incidence rate ratios (IRRs) for OHLC were calculated relative to IHLC as the reference. The values of IRR greater or lower than 1 indicate higher exophagy relative to the reference group.

The proportion of exposure experienced indoors by community members was calculated using methodology from Seyoum et al. [31]. Estimates were derived for the proportion of exposure to bites occurring while people were indoors both with (π_i,n_) and without (π_i_) LLIN use, assuming personal protection from LLINs was 93.7% as estimated from net studies [31]. In each village, the number of anophelines of each species captured per hour of the night both indoor and outdoor was combined with observational data estimating the proportion of the population inside/outside the households in each hour. This proportion was estimated from observational transect walks (see below) by calculating the mean number of people outside at each hour and adjusted for by the proportion of the village covered by the transect walks (estimated by counting the total number of household members in the households passed on the transects, divided by the total village population, from household census records).

### Observational transect walks

**Fig. 3:**
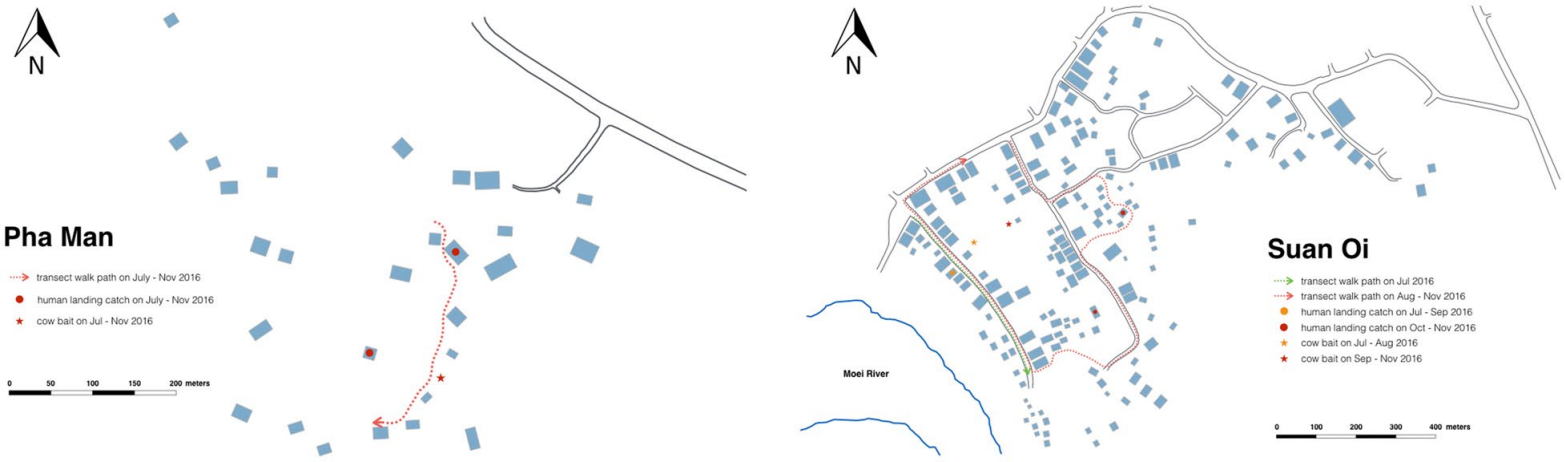
Transect walk routes. Map of Pha Man hamlet (left) and Suan Oi village (right) with sites of mosquito collections and route of transect walks

Concurrent to mosquito collections from July to November, transect walks were conducted through the study villages to observe the number of people outside their households at each hour of the night. The transects were walked on the hour, every hour from 18:00 to 06:00 via the routes in Fig. 3. Data were analysed to ascertain the mean number of people observed per hour per night, as well as what activities were being conducted by the community during these times. Data also fed into entomological data analyses as described in the previous section.

### Ethical clearance

This project was approved by Mahidol University and WHO TDR Ethics committee (Project ID B40443). All volunteers who carried out mosquito collections and GPS tracking signed informed consent forms and were regularly monitored for malaria infection by microscopy and for any malaria-like symptoms. House owners also gave permission to use their houses for collection of mosquitoes, blood surveys, direct observations and in-depth interviews.

## Results

### Behavioural and net coverage survey

There were 430 adults interviewed from 192 households in the three study villages; 57% of interviewees were female, 73% were Myanmar nationals and 95% were ethnic Karen. Most worked as general labourers (33%) or farmers (31%) and a large proportion of women were housewives (Additional file 1: Table S1). The 183 individuals that were identified as heads of households were asked about children aged under 18 years. From these 183 households, there were a total of 127 children aged under 5 years and 281 aged 5–17 years.

#### Household net coverage and use in village hamlets

Among households, ownership of at least one net of any type was 100% and ownership of at least one LLIN was over 90%; however, ownership of sufficient nets (one net per two people in the household), ITNs and LLINs was much lower (Table 2). There were 58.9% [95% CI 51.5–65.9] of households with sufficient ITNs and 57.8% [95% CI 50.5–64.9] with sufficient LLINs. Figures were lower in Pha Man (35.0% and 30.0% respectively), although CIs between villages overlapped. A total of 498 nets were owned among the 192 households. Of these, 87 (17.5, 95% CI [14.2–21.1]) were conventional untreated nets, three were conventionally-treated ITNs (0.6%, 95% CI [0.1–1.8]) and 408 (81.9%, 95% CI [78.3–85.2]) were LLINs; no hammock nets were owned.

**Table 2:** Coverage and utilization of nets and IRS among sampled households in each study community

	Komonae		Suan Oi		Pha Man		Total	
	n	% (95% CI)	n	% (95% CI)	n	% (95% CI)	n	% (95% CI)
Of households (N = 192)								
Households with at least one								
Any net	55	100 (93.5–100)	117	100 (96.9–100)	20	100 (83.2–100)	192	100 (98.1–100)
ITN	54	98.2 (90.3–100)	106	90.6 (83.8–95.2)	20	100 (83.2–100)	180	93.8 (89.3–96.7)
LLIN	54	98.2 (90.3–100)	105	89.7 (82.8–94.6)	20	100 (83.2–100)	179	93.2 (88.7–96.3)
Households with sufficient								
Any net	38	69.1 (55.2–80.9)	88	75.2 (66.4–82.7)	9	45.0 (23.1–68.5)	135	70.3 (63.3–76.7)
ITN	34	61.8 (47.7–74.6)	72	61.5 (52.1–70.4)	7	35.0 (15.4–59.2)	113	58.9 (51.5–65.9)
LLIN	34	61.8 (47.7–74.6)	71	60.7 (51.2–69.6)	6	30.0 (11.9–54.3)	111	57.8 (50.5–64.9)
Households that received IRS in previous 12 months	55	100 (93.5–100)	60	52.6 (43.1–62.1)	20	100 (83.2–100)	135	71.4 (64.4–77.8)
Households with sufficient LLINs and/or IRS in previous 12 months	55	100 (93.5–100)	92	78.6 (70.1–85.7)	20	100 (83.2–100)	167	87.0 (81.4–91.4)
Of individuals aged 18+ years (N = 430)								
% population access to an ITN		84.4 (78.3–90.4)		79.8 (73.9–85.7)		74.1 (63.1–85.2)		80.5 (76.4–84.6)
Adults that slept under a net previous night								
Any net	102	88.7 (81.4–93.8)	251	94.7 (91.3–97.1)	49	98.0 (89.4–99.9)	402	93.5 (90.7–95.6)
ITN	99	86.1 (78.4–91.8)	203	76.6 (71.0–81.6)	40	80.0 (62.3–90.0)	342	79.5 (75.4–83.3)
Use:access ratio		1		1		1		1
Of individuals aged < 18 years								
Children aged 5–17 slept that under LLIN previous night (N = 281)	66	94.3 (86.0–98.4)	129	77.7 (70.6–83.8)	38	84.4 (70.5–93.5)	233	82.9 (78.0–87.1)
Children aged < 5 slept under a LLIN previous night (N = 127)	25	96.2 (80.4–99.9)	66	80.5 (70.3–88.4)	14	73.7 (48.8–90.9)	105	82.7 (75.0–88.8)

*ITN* insecticide treated net, *LLIN* long-lasting insecticidal net, *IRS* indoor residual spraying

All households in Komonae and Pha Man reported having received IRS within the previous 12 months, along with half of households in Suan Oi. This high IRS coverage meant that although optimal LLIN coverage had not been achieved, 87.0% [95% CI 81.4–91.4] of households were protected with either LLINs or IRS, including all households in Komonae and Pha Man hamlets.

Despite lack of sufficient ITNs in the households, utilization of an ITN the previous night among the interviewed participants was high at about 80% and population access to an ITN was 80.5% (Table 2). The population use:access ratio was 1 across all villages. There were no differences in ITN utilization between villages or by gender, but utilization amongst Myanmar nationals was higher than Thai nationals (Additional file 2: Table S2). The few participants that did not sleep under a net (n = 28) stated that either they did not own enough nets (n = 4), using a net was too hot (n = 5), they did not like using a net (n = 18) or they found a net uncomfortable to breathe under (n = 3). Heads of households reported high LLIN use among children aged < 18 years at over 80% across all villages.

#### Farm hut and forest going and net use

One quarter of households owned a farm plot, highest in Pha Man compared to Komonae and Suan Oi (Table 3). The majority (67.4%) of farm plots were situated on the Myanmar side of the border and in Pha Man, 22.2% of households had farm plots on both the Thailand and Myanmar side of the border. A total 22.7%, 95% CI [18.8–26.9] of participants occasionally stayed overnight on a farm plot, with 49.5% of these staying once every week or 2 weeks, and 32.6% staying more than once per week. Among people sleeping at the farm huts, 63.5%, 95% CI [53.1–73.1] used a net (of any type) last time they stayed there, particularly high among those from Suan Oi village (92.6%). Repellent spray was used by 97.9%, 95% CI [92.7–99.7] of people last time they slept at the farm and 11.3%, 95% CI [5.8–19.4] wore long clothes to protect from mosquito or insect bites.

**Table 3:** Farm hut ownership and overnight stays, risk behaviours and use of personal protection among adults aged 18+ years in the three study villages

	Komonae		Suan Oi		Pha Man		Total	
	n	% (95% CI)	n	% (95% CI)	n	% (95% CI)	n	% (95% CI)
*Per household as reported by HH head (N = 192)*								
HH owns a farm plot	17	30.9 (19.1–44.8)	21	17.9 (11.5–26.1)	9	45.0 (23.1–68.5)	47	24.5 (18.6–31.2)
Country in which farm is located								
Thailand	1	5.9 (0.1–28.7)	8	40.0 (19.1–63.9)	4	44.4 (13.7–78.8)	13	28.3 (16.0–43.5)
Myanmar	16	94.1 (71.3–99.9)	12	60.0 (36.1–80.9)	3	33.3 (7.5–70.1)	31	67.4 (52.0–80.5)
Both	0		0		2	22.2 (2.8–60.0)	2	4.4 (0.5–14.8)
*Per individual adult (N = 430)*								
Sometimes stay overnight on a farm plot								
Yes	48	41.7 (32.6–51.3)	28	10.6 (7.2–15.0)	21	42.0 (28.2–56.8)	97	22.7 (18.8–26.9)
No	67	58.3 (48.7–67.4)	235	89.4 (85.0–92.8)	29	58.0 (43.2–71.8)	331	77.3 (73.1–81.2)
Frequency of staying overnight on farm								
> Once per week	11	22.9 (12.0–37.3)	19	70.4 (49.8)	1	5.0 (0.1–24.9)	31	32.6 (23.4–43.0)
Weekly-once per 2 weeks	32	66.7 (51.6–79.6)	6	22.2 (8.6–42.3)	9	45.0 (23.1–68.5)	47	49.5 (39.1–59.9)
Once per month	2	4.17 (0.5–14.3)	1	3.7 (0.09–19.0)	1	5.0 (0.1–24.9)	4	4.2 (1.2–10.4)
Farming season only	3	6.3 (1.3–17.2)	1	3.7 (0.09–19.0)	9	45.0 (23.1–68.5)	13	13.7 (7.5–22.3)
Used a net last time stayed overnight on farm								
Yes	22	45.8 (31.4–60.8)	25	92.6 (75.7–99.1)	14	66.7 (43.0–85.4)	61	63.5 (53.1–73.1)
No	26	54.2 (39.2–68.6)	2	7.4 (0.9–24.3)	7	33.3 (14.6–57.0)	35	36.5 (26.9–46.9)
Used other prevention method last time								
Long clothes	2	4.2 (0.5–14.3)	7	25.0 (10.7–44.9)	2	9.5 (1.2–30.4)	11	11.3 (5.8–19.4)
Treated clothing	0		4	14.3 (4.0–32.7)	0		4	4.1 (1.1–10.2)
Spray	48	100 (92.6–100)	27	96.4 (81.7–99.9)	20	95.2 (76.2–99.9)	95	97.9 (92.7–99.7)

Almost one-tenth of households with children aged 5–18 years stated that the children occasionally stay overnight on the farm, mostly staying once per month but one quarter visited more than once per week and most used a net the last time they stayed on the farm/forest. No data was gathered for this on children aged < 5 years. One-tenth of respondents stated that they occasionally stayed overnight in the forest (n = 48, Table 4), significantly lower in Suan Oi (n = 7; 2.6, 95% CI [1.1–5.4]) compared to Pha Man (n = 12; 24.0%, 95% CI [13.1–38.2]).

**Table 4:** Forest-going activities and use of personal protection among adults aged 18+ years in the three study villages

	Total (N = 48)
	% (95% CI)
Stay overnight in the forest	11.2 (8.3–14.5)
Frequency of staying in forest	
> Once per week	12.5 (4.7–25.2)
Once week/once 2 weeks	68.8 (53.7–81.3)
Once month	12.5 (4.7–25.2)
Rarely	6.3 (1.3–17.2)
Reason for going to forest	
Foraging	87.5 (74.8–95.3)
Hunting	91.7 (80.0–97.7)
Other (goat farming, fishing)	4.2 (0.5–14.3)
Forest is in which country?	
Thailand	14.6 (6.9–28.2)
Myanmar	47.9 (33.8–62.3)
Both	37.5 (24.7–52.4)
Used net last time stayed in forest	
Yes	9.5 (3.5–23.6)
Used other prevention method last time in forest	
Long clothes	87.5 (74.2–94.4)
Repellent spray	47.9 (33.8–62.3)

Most would stay overnight in the forest at least fortnightly or more in order to hunt or forage for forest products. Only 4 forest-goers (9.5%, 95% CI [3.5–23.6]) used a net (of any type) the last time they stayed in the forest, all were from Suan Oi village. Most respondents wore long clothes to protect from mosquito bites, and around half used repellent spray.

#### Sleeping times

**Fig. 4:**
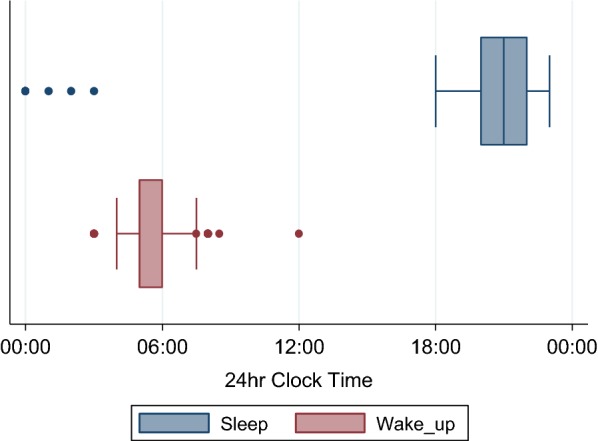
Sleeping and waking times of community members. Box plots show median, interquartile range (IQR) and range of sleeping and waking times among respondents aged ≥ 18 years

The median time for sleeping among respondents was 21:00 h, although the range of sleeping times was very wide, from 18:00 till 03:00 considering all outliers in the population (Fig. 4). The median waking time for adults was 05:00 but again, the range was very wide, with some people waking from 03:00.

### Entomological results

#### Anopheles abundance, exophagy and zoophagy

A total of 3636 anopheles mosquitoes were captured from a total of 103 collection nights of HLC and cow-baited collections. Of these, 75.3% were the primary vectors—*An. dirus* (0.4%, n = 13), *An. maculatus* (61.6%, n = 2238) and *An. minimus* (13.3%, n = 483); and 11.6% were the secondary vectors—*Anopheles annularis* (4.5%, n = 162), *Anopheles barbirostris* (0.6%, n = 24), and *Anopheles culicifacies* (6.5%, n = 235).

Human biting rates were highest in the farm hut sites where all three primary vector species were found—*An. dirus* 0.30 bites per person per night (bpn), *An. maculatus* 4.25 bpn and *An. minimus* 5.78 bpn (Table 5)—as well as all three secondary vectors (0.31 bpn collectively).

**Table 5:** Multivariate analysis of mosquito collection methods associated with abundance of three primary vector species by catch site

	*An. dirus*		*An. maculatus*			*An. minimus*		
	bpn (n)	IRR^a^ (95% CI)	bpn (n)	IRR^a^ (95% CI)	p value	bpn (n)	IRR (95% CI)	p value
Suan Oi								
HLC indoor	0	–	0	–	–	0.08 (1)	1.37 (0.19, 9.85)	0.76
HLC outdoor	0	–	0	–	–	0.33 (4)	0.91 (0.33, 2.50)	0.86
Pha Man								
HLC indoor	0	–	1.67 (20)	1		5.67 (68)	1	
HLC outdoor	0.17 (2)	–	2.58 (31)	1.47 (0.84, 2.58)	0.18	4.25 (51)	0.71 (0.49, 1.02)	0.07
Farm huts								
HLC	0.30 (10)	–	4.21 (139)	1.53 (0.96, 2.45)	0.08	5.63 (186)	0.60 (0.46, 0.80)	< 0.001

Multivariate analysis conducted using negative binomial regression and on the average bites per person per night (bpn). Estimates are from HLC data only, bites per night adjusted for difference in frequency of extended hours of collection (17:00–18:00 and 06:00–07:00 collections conducted for 24/33 nights)

^a^Not enough data to calculate IRR. IRR: estimated incident rate ratio, CI: confidence interval, corresponding p-values based on maximum likelihood estimation of suitable Negative Binomial regression models

In Pha Man village outdoor biting rates of *An. dirus*, *An. maculatus.* and *An. minimus* were 0.17, 2.58 and 4.25 bpn, respectively (Table 5). *Anopheles minimus* had a greater tendency to bite indoors with an indoor biting rate of 5.67 bpn and an exophagic rate of 43%. In comparison, *An. maculatus* had a tendency to bite outdoors with a lower indoor biting rate of 1.67 bpn and an exophagic rate of 61%. No *An. dirus* were captured indoors. Of the secondary vectors, only *An. annularis* bit indoors but all three were found in the outdoor catch site, albeit with low biting rates (0.58 bpn collectively).

**Fig. 5:**
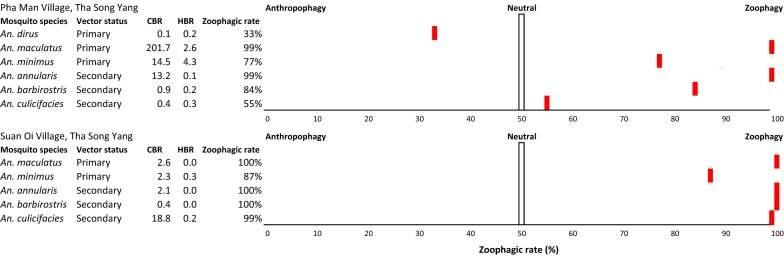
Zoophagy rates (%) of the primary and secondary vector species captured by cattle and outdoor human landing catch in the village study sites

Exposure to anopheles bites in Suan Oi village was much lower than in Pha Man hamlet and farm huts. Only one primary vector species was captured—*An. minimus*, with indoor and outdoor biting rates of 0.08 and 0.33 bpn, respectively, and an exophagic rate of 80%—as well as one secondary vector—*An. culicifacies* (outdoor biting rate 0.17 bpn). Only *An. dirus* showed a preference for human-biting in the village sites (zoophagic rate 33%, Fig. 5). *An. maculatus* and *An. minimus* showed a strong zoophagic preference with zoophagic ratios of 99% and 77%, respectively in Pha Man and 100% and 87% in Suan Oi.

Multiple Negative Binomial regression models were built separately for the 3 species using only the HLC data with IHLC in Pha Man hamlet site designated as the reference group. Table 5 summarizes the incidence rate ratios (IRR) from those models. Statistical analysis revealed that the biting rate of *An. minimus* was significantly higher in farm huts than IHLC in Pha Man hamlet (p < 0.0001). No IRRs were computed for *An. dirus* due to insufficient numbers.

#### Hourly biting profiles

**Fig. 6:**
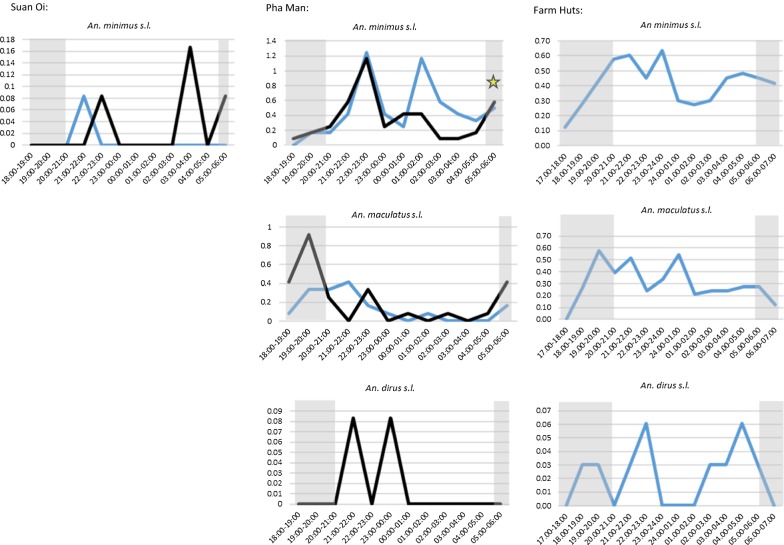
Hourly biting profiles of primary vector species captured by HLC. Plots show hourly biting rates of primary vector species caught by IHLC (blue line) and OHLC (black line) in three capture sites: Suan Oi village (left-hand column); Pha Man hamlet (middle) and Farm huts (right-hand column). Yellow star represents where a *Plasmodium*-positive sample was captured. Shaded grey area is time outside of median sleeping hours reported by adults in the cross-sectional survey

In Suan Oi village very few *An. minimus* were captured but these included captures at both 21:00–22:00 and late in the morning at 05:00–06:00 (Fig. 6). In Pha Man village where sample size was larger, the first biting peak was at 22:00–23:00 for both indoor (1.25 bpn, n = 15) and outdoor (1.17 bpn, n = 14) HLC, followed by a second indoor biting peak at 01:00–02:00 (1.17 bpn, n = 14). The biting rates for both IHLC and OHLC were also relatively high in the last hour of collection (05:00–06:00) and this was the period when a single *P. vivax*-infected *An. minimus* was captured (denoted by a star symbol in Fig. 6). In the farm huts peak biting from *An. minimus* started earlier, reaching 0.58 bpn at 20:00–21:00. The first *An. minimus* were also caught during the extended hours of collection at 17:00–18:00 (0.13 bpn, n = 3). Biting fell in the early morning but rose again from 03:00 and remained relatively high until the late morning 06:00–07:00 period.

*Anopheles maculatus* were caught in Pha Man and in the farm huts. In Pha Man, outdoor biting rates peaked early in the evening at 19:00–20:00 (0.92 bpn, n = 11) and then biting remained low except for smaller peaks at 22:00–23:00 (0.33 bpn, n = 4) and 05:00–06:00 (0.42 bpn, n = 5). Indoor biting rates remained lower except for a peak at 21:00–22:00 (0.42 bpn, n = 5) and the majority of indoor biting occurred before 22:00 (n = 14, 70% of all-night indoor biting). In the farm huts *An. maculatus* biting hit an early peak from 19:00–20:00 (0.58 bpn, n = 19) and hit similar peaks at 21:00–22:00 (0.52 bpn, n = 17) and 00:00–01:00 (0.55 bpn, n = 18).

Only two *An. dirus* were captured in Pha Man, one each at 21:00–22:00 and 23:00–0:00 (0.08 bpn), both outdoors. In the farm huts, the majority were caught at 22:00–23:00 (0.06 bpn, n = 2) and 04:00–05:00 (0.06 bpn, n = 2), but the first was caught during the 18:00–19:00 h period.

Using median sleeping and waking times reported by adults in the cross-sectional behavioural survey (grey shaded areas in Fig. 6), the proportion of all-night biting by primary vectors that occurred outside of the time when most people could reasonably be expected to use a net (before 21:00 and after 05:00) was 20.0% in Suan Oi, 33.7% in Pha Man and 37.6% in the farm huts (counting both IHLC and OHLC combined). Around one-tenth of bites were post-5 a.m. for all three primary vectors (13%, 10% and 8% of *An. minimus*, *An. maculatus* and *An. dirus* biting, respectively).

#### Differences between farm huts

**Fig. 7:**
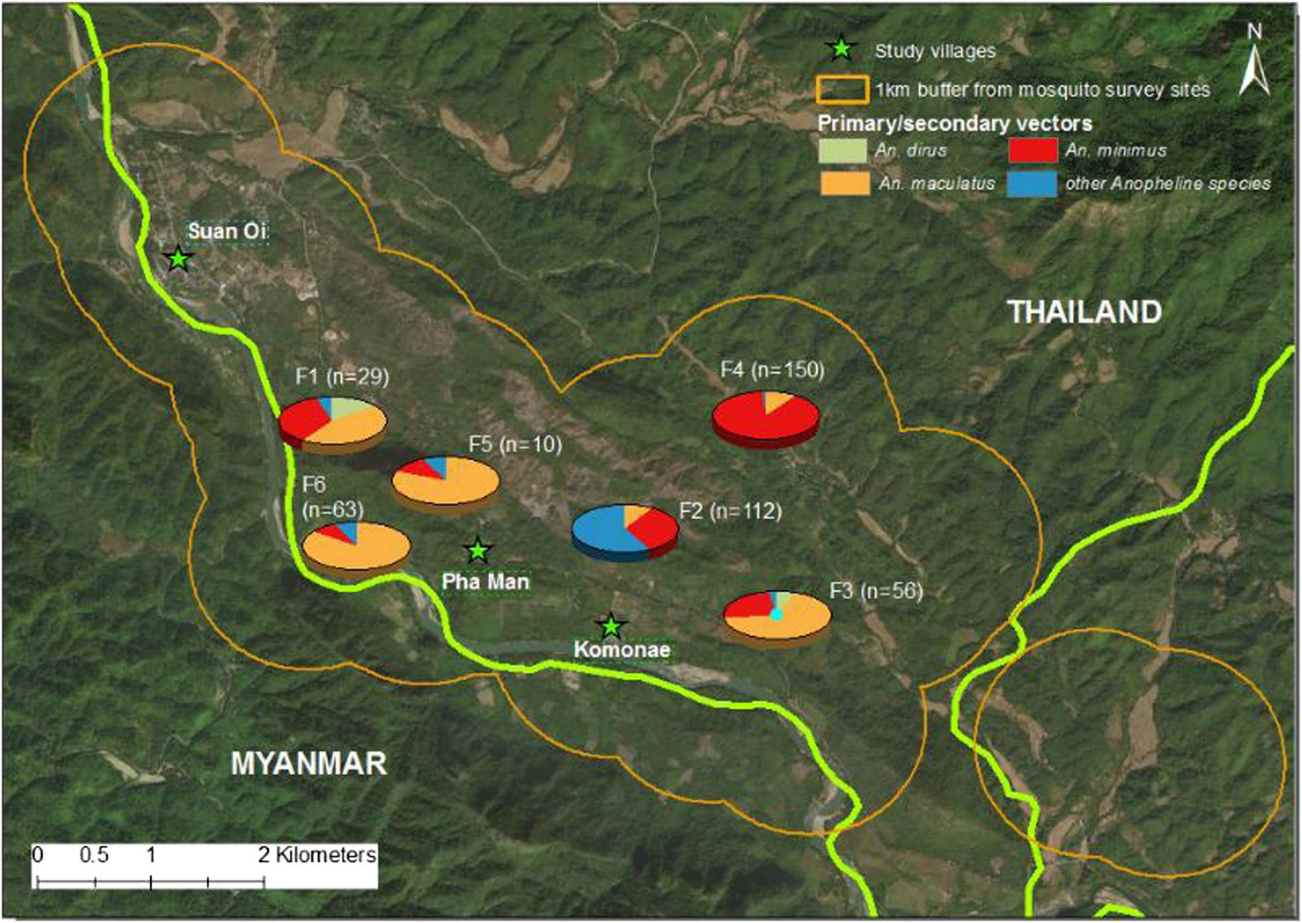
Map showing abundance and species composition in each of the forested farm hut collection sites. Pie charts each represent the location of one of the six forested farm hut collection sites, denoted F1–F6. Total number of anophelines captured in each location is shown in brackets while pie charts show the breakdown by species. Orange line represents a 1 km buffer zone around the six sites. Green stars show each study village and green line is the international border between Thailand and Myanmar

There were differences in anopheline abundance and species composition between forested farm hut sites (Fig. 7). Each forested location had 6-person nights of collection conducted, except F6 which has three and the number of anophelines captured ranged from just 10 (F5) to 150 (F4). In four of six sites, *An. maculatus* was the dominant species, while in F4 *An. minimus* dominated and in F2 secondary vector species were most dominant.

#### Infectivity

Of all 3636 anopheline mosquitoes tested by sporozoite assay, only one tested positive for *P. vivax* CSP PV210 by ELISA assay, giving a sporozoite index from all anophelines of 0.03%, 95% CI [7 × 10^−4^ − 0.15]. This specimen was *An. minimus* captured by OHLC in Pha Man village in August during the 05:00–06:00 collection. The EIR for *An. minimus* was thus 2.5 infective bites/person/month in the outdoor Pha Man catch site (and 0.4 infective bites/person/month outdoors in Pha Man considering all primary vectors) and zero for the other catch sites and anopheles species.

##### Transect walks

**Fig. 8:**
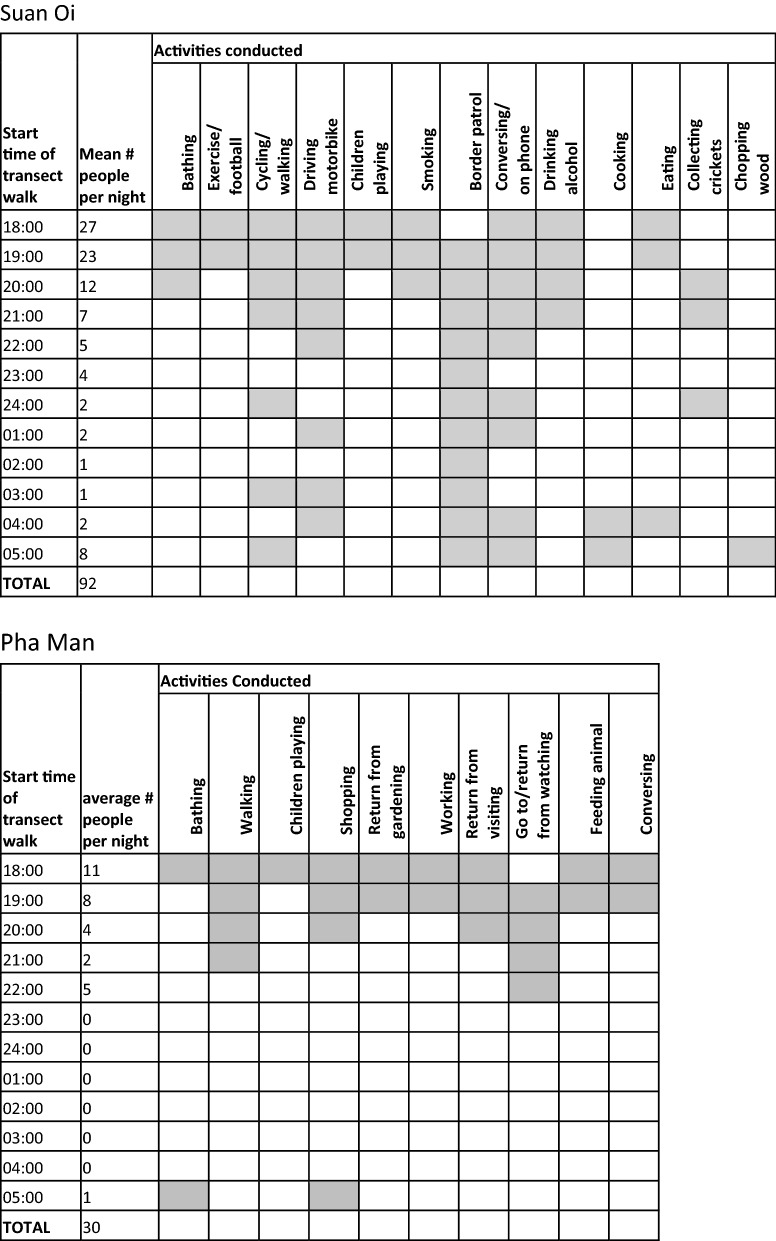
Activities conducted outside of the household (shaded squares) per hour of the night in Suan Oi (top) and Pha Man (bottom)

In Pha Man village, a total of 335 individuals were observed outside during the hours of 18:00–06:00 over 11 sampling nights (mean per night = 30.45; Fig. 8). In Suan Oi, 1109 individuals over 12 nights were observed outside (mean per night = 92.42; Fig. 8). Mostly people were observed conversing with each other outdoors, but other activities differed between villages with most other people in Pha Man travelling to and from households of relatives and friends or neighbours with televisions, while in Suan Oi there was a greater variety of activities including bathing, exercising, walking, and cycling as well as border guards on patrol (Fig. 8).

#### Proportion exposure occurring indoors

**Fig. 9:**
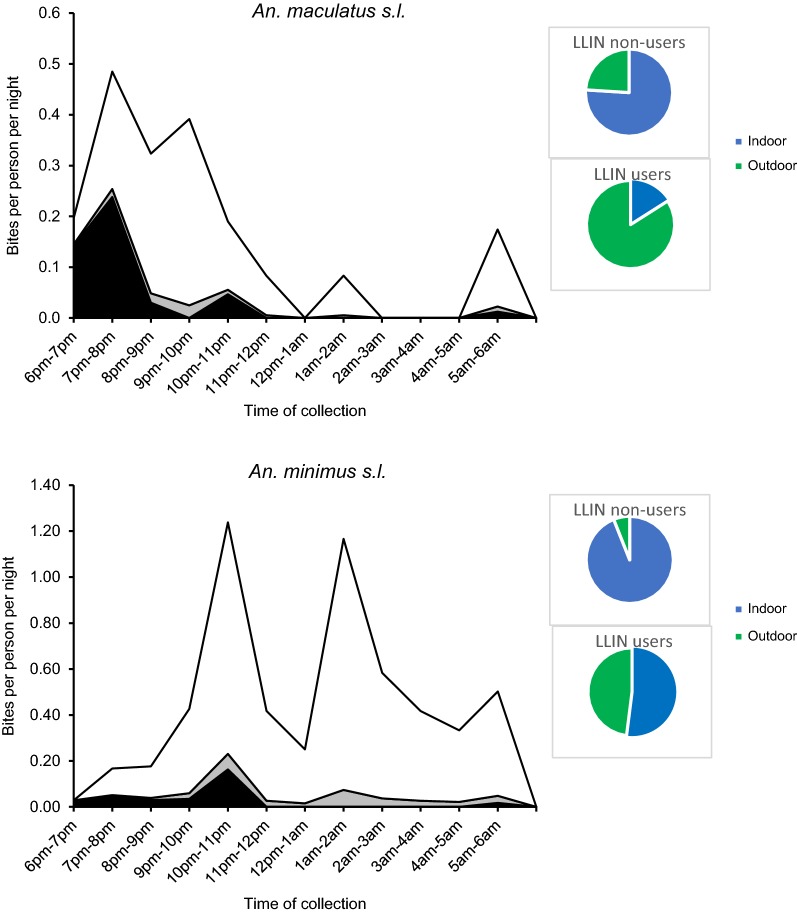
Indoor and outdoor biting exposure per hour of the night in Pha Man from *An. maculatus* and *An. minimus* for users and non-users of LLINs. Stacked lines show: indoor biting exposure for non-users of ITN (white area), indoor biting exposure for users of ITN (grey area) and outdoor biting exposure (black area). Pie charts show: proportion of total nightly biting exposure occurring indoors and outdoors for users and non-users of LLINs

It was estimated that the transect walks covered 26.7% of the population in Pha Man (n = 120) and 39.9% in Suan Oi (n = 222). These denominators were used to estimate the proportion of the population inside households at each hour of the night; however, since *An. maculatus* and *An. minimus* in Pha Man village were the only vectors that had both (relatively) high indoor and outdoor biting rates only these gave meaningful results (Fig. 9). *An. minimus* was captured mostly indoors and during the night time hours the majority of community members also resided indoors, therefore, exposure to *An. minimus* bites was higher inside households than outside for both users and non-users of LLINs (Fig. 9 and Additional file 3: Table S3). Even though the majority of *An. maculatus* were captured outdoors, the majority of human exposure to biting still occurred indoors from *An. maculatus* for non-users of LLIN (0.76). However, use of an LLIN meant the proportion of indoor exposure dropped to just 0.16. This resulted in hourly biting risk as shown in Fig. 9.

## Discussion

This mixed methods study provides evidence on potential factors contributing to, and modulating, sustained transmission in these rural communities of Thailand. There was clear variation in primary vector anopheline abundance and composition between ecological sites, including between individual villages, between villages and farm huts, as well as between different farm hut locations. When overall transmission reaches such low levels, the result is high heterogeneity even between closely neighbouring sites. High coverage of vector control and deforestation activities that have removed mosquito larval habitats mean vectors are limited to areas that retain their preferred ecology, in this case the forested settlements and farm huts. Unfortunately it was not feasible to quantitatively measure forest cover in each location to explore whether this could be a contributing factor to site heterogeneity. Deforestation may deplete populations of deep-forest vectors and so reduce malaria transmission; although, in some localities this depletion may be followed by the invasion of the deforested areas by other, less efficient, vectors and a potential increase in transmission again [32, 33]. With the exception of a longitudinal study examining the effects of progressive land use changes from pre-development forest to oil palm cultivation on the distribution of disease vectors and malaria incidence [34], there is a striking lack of primary research directly measuring the impact of deforestation on malaria in South-east Asia [32].

As well as having greater abundance of vectors, people were more exposed in the forested locations because they either slept in huts with open walls or in the open on the forest floor, and there was lower use of LLIN. A risk factor study in a community in Viet Nam, showed that wooden or bamboo houses had a higher risk for malaria infection compared to cement houses in the same village (odds ratio 4.18, 95% CI [1.45–12.10]) [35] and in Cambodia, open housing blurred the indoor/outdoor biting distinction [13]. Surveys conducted in Viet Nam showed that the risk of mosquito house entry was more than twice as high in traditional bamboo houses compared with those newly constructed from wood (putative Japanese encephalitis vector IRR = 2.26, 95% CI 1.38–3.70, p = 0.001; anopheline IRR = 2.35, 95% CI 1.30–4.23, p = 0.005) [36] and in Laos, risk of house entry by anophelines was more than twice as high in traditional bamboo houses compared with those newly constructed from wood (anopheline IRR = 2.35, 95% CI 1.30–4.23, p = 0.005) [36]. Building houses from straight-edged wooden slats probably reduces the number of gaps in the walls and floors of a house through which a mosquito might enter, compared with open bamboo housing which is likely to have many more holes through which mosquitoes could enter and out of which host odours could pass. Promoting the use of bed mats for people sleeping under bed nets and improving housing conditions would both likely increase protection from malaria [37].

Deficits in LLIN coverage increased exposure risk at the village and, to a greater extent, in forested locations. Despite reports to the contrary during site selection, coverage of LLINs in the villages was not universal. Although the percentage of households owning sufficient LLINs was 58% overall and only 30% in Pha Man, high coverage of IRS meant most households were protected by vector control. Furthermore, reported utilization of ITN/LLIN the previous night and population access to an ITN were around 80% for all villages. Despite the tendency for many vectors to bite outside, maintaining high LLIN coverage and use has been demonstrated to be essential to achieve malaria elimination and prevent re-establishment of transmission [38, 39]. Although estimates of the proportion of the population indoors at each hour were crudely estimated from transect walk data, results suggest that there is a higher proportion of biting exposure that occurs indoors than outdoors for non-users of LLINs despite anophelines being exophagic. When people used LLINs, the proportion of exposure occurring indoors fell substantially. Similar results were observed in western Kenya where LLIN-users experienced a moderate reduction in their overall exposure to endophagic primary vector species (*Anopheles funestus* s.l. and *Anopheles arabiensis*) and an exophagic vector (*Anopheles coustani*) from 1.3 to 0.47 bpn, resulting in a 51% protective efficacy of nets varying significantly with age and season (p < 0.01) [40]. Therefore, efforts need to be made to maintain high LLIN coverage and utilization in these communities even in the face of dwindling malaria cases, which can affect community practices due to low perception of risk [41, 42]. A recent modelling study showed significant short-term reduction of mosquito populations and EIR due to combinational intervention of 50% LLINs plus outdoor Attractive Toxic Sugar Baits (ATSBs) compared to 50% LLINs alone in an African village with clustered houses, followed by increased probability of local mosquito extinction at the time when annual EIR is less than one per person [43].

The gap in LLIN access and usage, however, appears when community members move into the farm huts and forest without carrying or using a bed net. LLIN distribution programmes need to be able to account for communities with dual residence systems. The village tends to be the lowest unit of focus for control programmes and research activities, although more often control is focused at even higher administrative levels such as the sub-district or district level. Case-based surveillance and focus investigations for elimination generally target a patient’s home village rather than considering the most likely transmission site. Yet results show a need to look even beyond the village to the farm huts and forest locations that are frequently visited by community members since these have the higher abundance of anophelines and highest risk practices. Over 40% of people in Komonae and Pha Man slept overnight at farm huts and 25% slept in the forest, and of these, around one-third of adults did not use a net in the farm huts and over 90% did not use a net in the forest. Since type of net and quality of net were not reported or observed it may be that these numbers are lower for those using an effective LLIN. It may be possible for the proportion using an LLIN in the farm huts to be increased through additional LLIN distribution and education of users, although the size and structure of huts may be a hindrance to effective net use, either because, as described above, anophelines are able to enter semi-open huts [44], or nets may be used for nuisance protection during the day [45, 46], or there may be difficulty involved with frequently carrying a net between the main residences and farming huts [47]. Programme planners need to take the extra nets used in farming huts into account when calculating the number required for distribution and consider the most appropriate net features for the local situation. Furthermore, attention should be paid to bed net attrition and durability through strengthening of pre- and post- bed net distribution surveys [48, 49].

It would not be possible for forest goers to use a traditional LLIN in the forest since there is no reasonable place for them to hang it, an issue which long-lasting hammock nets (LLIHNs) are designed to overcome. However, no hammock nets were reported to be owned by the households surveyed. It is not clear whether this was due to a lack of availability or affordability, or whether deemed unnecessary or useless if, for example, people are moving around during the night-time working or hunting. Attitudes towards use of hammocks appears to vary across the GMS with evidence of higher use in Vietnam and Cambodia than Myanmar [50–52]. In situations where hammocks are not utilized by the local population, alternative personal protection methods would be required. Repellent use among forest goers in the current study was very high, suggesting some desire for personal protection. However, repellents have been shown to be ineffective against mosquito biting mostly due to poor adherence to reapplication of the spray [53]. Given the popularity and cultural acceptance of thanaka and DEET (di-methyl benzamide) in the local Karen community [54, 55], there is a need to evaluate the ‘protective’ effectiveness of this tool as a supplement to traditional indoor practices for malaria elimination, especially in village settings of clustered houses where LLINs alone is far from sufficient.

Even with use of effective LLIN during sleeping hours, risk of exposure to indoor biting remained high, particularly in Pha Man because there was a high proportion of biting that occurred outside of the median sleeping times of 21:00–05:00. Primary vector biting started early in the evening from 18:00 and lasted until at least 06:00 in the village and 07:00 in the farm huts when collections were terminated. This is similar to patterns seen in Cambodia where biting in the village lasted until 8 a.m. albeit at low levels [13]. This Cambodia study collected data on the difference in sleeping times between village and farms, with farm hut sleeping times being dictated by the sun due to lack of electricity. Although data on this was not collected here, the same is likely true of the study sites, however any potential reduction in exposure this could bring if people were to use nets may be counteracted by the even earlier evening and later morning biting rates from the primary vector species in the farm huts compared to the village settings. This highlights a limiting factor in use of nets and an area where personal protection methods would need to be applied.

There was also increased risk of biting exposure through human behaviour that took people outside of their households in the evening and early morning. A small proportion of the community were observed to be outside during all hours of the night, particularly in Suan Oi where there were border patrol activities and where electricity is more commonly available. In Pha Man electricity is limited and thus people tend to be inside households during the dark night time hours. Indeed, one of the key activities taking people outside in this hamlet was the fact that people would walk to a neighbour’s house to watch TV before going back home to sleep. The small proportion of the community who stay outside for longer periods of the night are at greater risk of biting since the majority of vectors in each environment showed a tendency for outdoor biting between 18:00 and 22:00. The preference of the primary vectors to bite both outdoors as well as on animal bait may lower the direct risk to humans but it also means that they avoid the effects of the insecticides in nets and IRS, lowering the community effect of insecticides on mosquito populations [56–58]. However, these preferences could also be exploited for vector control through treatment of cattle or other livestock or their surroundings with, for example, transmission-blocking drugs (e.g. ivermectin) or agents that kill mosquitoes or prevent their successful breeding (e.g. pyriproxyfen dust) [59–61].

Despite low anopheline abundance, health facility records showed Suan Oi had the highest human malaria incidence of the whole district. It is not immediately clear why from the data—Suan Oi had a lower proportion of households protected by either sufficient LLINs or IRS, but an equal proportion of the population using nets and a lower proportion of people sleeping overnight in the farm hut or forest. Those that did sleep at the farm or forest were also more likely to use a net while there. Contributors to this that were unable to be explored could be population differences in immunity to clinical malaria episodes, differences in healthcare-seeking and treatment uptake, and *P. vivax* relapse rates.

Differences in immunity could occur between the village populations which can be affected by the extent and frequency of exposure as well as genetic factors [62]. In terms of healthcare seeking, Pha Man does not have a health centre located within it and the population are noticeably less wealthy than those in Suan Oi. In other Asian settings, healthcare seeking can be reduced in more rural settings with further distance to the nearest facility and lower wealth [63, 64], although in the current study area it was previously shown that these did not have a significant affect and other factors such as ethnicity and social support can play a vital role [65]. *Plasmodium vivax* was the only parasite detected in recent prevalence surveys of the area and therefore some of the infections could be from relapse of previous infections. In central Viet Nam, relapse cases were shown to have a 59% chance of having parasitaemia for 4 months or longer [66]. Persistent and largely asymptomatic *P. vivax* (and *P. falciparum*) infections are common in many areas of low seasonal malaria transmission [66, 67] and infections with low-density parasitaemia can develop into much higher density infections at a later time, which are likely to sustain RMT and endemicity. In Papua New Guinea, *P. vivax* relapses cause approximately 50% of infection and more than 60% of clinical episodes in the first 3 months of follow-up, though with little effect thereafter [68], and the mean number of relapses per infection is 4.3, 95% CI [4.0, 4.6] over a 16 month period for a 3-year old child [69].

Another aspect which could not be explored in this study, but which forms a vital part of the definition of RMT, is that of insecticide resistance. An issue of concern in the villages is the overlap of pyrethroid-based LLINs and IRS. The two interventions together are not recommended by WHO since they can select for pyrethroid-resistance [70]. As the existing definition of RMT requires that mosquitoes must be susceptible to these tools, the status of insecticide resistance in the primary and secondary vectors in Tha Song Yang is unknown. Although most *Anophele*s species in Thailand remain susceptible to insecticides, pyrethroid resistance has been found in northern Thailand (Chiang Mai) where significant amounts of pesticides are used for agricultural pest control [71, 72]. Resistance or suspected resistance to pyrethroids was detected in primary (*An. minimus, An. maculatus*) and secondary vectors (*An. barbirostris*, *Anopheles hyrcanus*) on the Myanmar side of the Thai–Myanmar border neighbouring Mae Hong Song province, Thailand [73]. Indeed, geographical variations in insecticide resistance occur among potential malaria vectors and in *An. minimus* in Ubon Ratchathani and in Chiang Mai (both in north-eastern Thailand), respectively [71, 74]. Documenting the susceptibility to public health insecticides is important in the framework of RMT and elimination.

Although the results offer some important insight into the various factors contributing to RMT in these study communities, the study is limited in its characterization of the farms and forested areas frequented by the community members since many of them were over the border in Myanmar and were thus inaccessible to the survey team. Although the local population moves frequently across the border, the study was limited to the Thailand side where approvals were in place and thus, may potentially have missed the higher transmission zones. Furthermore, due to logistical constraints and being the first time that mosquito collections have been attempted beyond the village, the farm hut sites were not collected concurrently to the villages and thus numbers may not be entirely comparable. Having proved the feasibility of sampling beyond the village it is hoped that future expansion of the study could improve upon the sampling methods, as well as look at characterizing a greater number of sites, including across the border.

It is possible that results were also affected by the El Niño in 2016 which meant average rainfall was lower, thereby potentially decreasing the mosquito population and malaria incidence [75, 76]. This would most likely mean mosquito populations have been underestimated and it may help explain why only one *Plasmodium* sporozoite-positive specimen was collected. Data showed that the overall sporozoite rate (0.03%) was 37-times lower than that found in four villages 50 km away from the study site along the Thai–Myanmar border [8]. Although conclusions cannot be made about seasonality of transmission since the collection period did not span the dry season, the study sites have a minor peak in malaria incidence from September to November during the transition from wet to dry season [19], which coincided here with the presence of all three major *Anopheles* species. Other longitudinal studies have found marked seasonal differences in trophic behaviour and biting activity of primary vector species [8, 23, 77, 78].

Finally, it is likely there are a multitude of other factors that could be contributing to RMT in these areas that were out of the scope of this study, including anopheline sibling species composition, *Plasmodium* species composition and carriage of asymptomatic infection, and climatic variables. Although one of the three major vector species, *An. minimus,* was found to be infective in this study and *An. annularis* and *An. barbirostris* have previously been found to be infective in this study area [19], it is unclear which species are the main drivers of RMT in the study area. The abundance of biting in relatively hard-to-reach farm huts and forested locations in which individuals are less well protected and at risk from crepuscular biting, will contribute to asymptomatic carriage of infection and thus potentially sustain transmission through undetected infections. Recent epidemiological analysis show that *P. vivax* cases were significantly associated with mosquito capture rates and less with migrant status, indicating local transmission compared to *P. falciparum* infections which occur mostly in the recent migrant population with a seasonality reflecting that of agricultural activity, rather than that of the local mosquito population [18]. These transmission characteristics are representative of the area in terms of environment, ecology, population, and behaviour, particularly that of cross-border migration, populations of which serve as an important reservoir for malaria transmission in Thailand [8, 79]. This transmission makes interventions like ivermectin, impregnated clothing and topical repellents even more relevant.

## Conclusion

This study has highlighted some potential contributors to RMT in these communities. The factors analysed here are relatively open to programmatic intervention and thus could be used to inform future strategies for elimination in the region. Future work could be done to extend these methods within this study region and to others where contributing factors may be different. Novel personal protection tools that require minimal behaviour change and that are accessible/affordable for the target populations such as, treated blankets, treated clothing, spatial and topical repellents, need to be tested and implemented in order to fill the gaps in protection from LLIN and IRS. Given the low transmission setting, use of epidemiological endpoints in randomized control trials of these tools would be unfeasible and thus the use of entomological endpoints to prove efficacy needs to be accepted among donors, governments and regulatory bodies if the utility of such tools in the elimination context is to be realized.

## Additional files

**Additional file 1: Table S1.** Demographics of the surveyed adult (aged 18 years and over) population in the three study villages of Tha Song Yang district.

**Additional file 2: Table S2.** Utilization of bed nets the previous night by demographic factor among the surveyed adult population.

**Additional file 3: Table S3.** Calculation of indoor and outdoor exposure risk for users and non-users of LLINs in Pha Man.

## Data Availability

The data that support the findings of this study are available from Malaria Consortium, but restrictions apply to the availability of these data, which were used under strict data restrictions approved by the relevant ethics bodies used in the current study, and so are not publicly available. Data are however available from the authors upon reasonable request and with permission of Malaria Consortium.

## Abbreviations

bpn: bites per person per night
CBR: cattle biting rate
CI: confidence interval
CS: circumsporozoite protein
EIR: entomological inoculation rate
GMS: Greater Mekong Subregion
HBR: human biting rate
IHLC: indoor human landing catch
IRR: incidence rate ratio
IRS: indoor residual spraying
ITN: insecticide treated net
LLIN: long-lasting insecticidal net
NMCP: National Malaria Control Programme
OHLC: outdoor human landing catch
PBS: phosphate-buffered saline
RMT: residual malaria transmission
VBDC: vector borne disease control

## References

[1] WHO Global Malaria Programme. Insecticide-treated Mosquito Nets: a WHO Position Statement. 2007. http://www.ivcc.com/sites/ivcc.mrmdev.co.uk/files/content/itnspospaperfinal.pdf. Accessed 7 June 2019.

[2] Hosking A. Efficacy of insecticide treated nets in South East Asia. In: Meek S, Kaviratne M, editors. Malaria Consortium annotated bibliography. 2010. p. 1–44.

[3] Kolaczinski J, Macdonald M, Meek S (2014). Vector control to eliminate artemisinin resistant malaria in the Greater Mekong Subregion. Lancet Infect Dis..

[4] Sochantha T, Hewitt S, Nguon C, Okell L, Alexander N, Yeung S (2006). Insecticide-treated bednets for the prevention of *Plasmodium falciparum* malaria in Cambodia: a cluster-randomized trial. Trop Med Int Health..

[5] Luxemburger C, Perea WA, Delmas G, Pruja C, Pecoul B, Moren A (1994). Permethrin-impregnated bed nets for the prevention of malaria in schoolchildren on the Thai–Burmese border. Trans R Soc Trop Med Hyg..

[6] Hii J, Rueda LM (2013). Malaria vectors in the Greater Mekong Subregion: overview of malaria vectors and remaining challenges. SE Asian J Trop Med.

[7] Smithuis FM, Kyaw MK, Phe UO, van der Broek I, Katterman N, Rogers C (2013). Entomological determinants of insecticide-treated bed net effectiveness in Western Myanmar. Malar J.

[8] Kwansomboon N, Chaumeau V, Kittiphanakun P, Cerqueira D, Corbel V, Chareonviriyaphap T (2017). Vector bionomics and malaria transmission along the Thailand–Myanmar border: a baseline entomological survey. J Vector Ecol..

[9] St. Laurent B, Miller B, Burton TA, Amaratunga C, Men S, Sovannaroth S (2015). Artemisinin-resistant *Plasmodium falciparum* clinical isolates can infect diverse mosquito vectors of Southeast Asia and Africa. Nat Commun..

[10] WHO Global Malaria Programme. Artemisinin and artemisinin-based combination therapy resistance. Status Report. Geneva: World Health Organization; 2017. https://apps.who.int/iris/bitstream/handle/10665/255213/WHO-HTM-GMP-2017.9-eng.pdf;jsessionid=C776905C7E5F82DA454900D72C402ADE?sequence=1. Accessed 7 June 2019.

[11] Erhart A, Ngo DT, Phan VK, Ta TT, Van Overmeir C, Speybroeck N (2005). Epidemiology of forest malaria in central Vietnam: a large scale cross-sectional survey. Malar J.

[12] Thang ND, Erhart A, Speybroeck N, Hung LX, Thuan LK, Hung CT (2008). Malaria in central Vietnam: analysis of risk factors by multivariate analysis and classification tree models. Malar J..

[13] Gryseels C, Durnez L, Gerrets R, Uk S, Suon S, Set S (2015). Re-imagining malaria: heterogeneity of human and mosquito behaviour in relation to residual malaria transmission in Cambodia. Malar J.

[14] Killeen GF (2014). Characterizing, controlling and eliminating residual malaria transmission. Malar J.

[15] Killeen GF, Kiware SS, Okumu FO, Sinka ME, Moyes CL, Massey NC (2017). Going beyond personal protection against mosquito bites to eliminate malaria transmission: population suppression of malaria vectors that exploit both human and animal blood. BMJ Glob Health..

[16] Ministry of Public Health. National Malaria Elimination Strategy Thailand 2017–2026 (in Thai). Bangkok, 2016. http://malaria.ddc.moph.go.th/downloadfiles/Malaria_Manual/%5BStrategy%5D_National_Malaria_Elimination_Strategy_Thailand_2017-2026.pdf. Accessed 7 June 2019.

[17] Jitthai N (2013). Migration and malaria. Southeast Asian J Trop Med Public Health.

[18] Sriwichai P, Karl S, Samung Y, Kiattibutr K, Sirichaisinthop J, Mueller I (2017). Imported *Plasmodium falciparum* and locally transmitted *Plasmodium vivax*: cross-border malaria transmission scenario in northwestern Thailand. Malar J.

[19] Sriwichai P, Samung Y, Sumruayphol S, Kiattibutr K, Kumpitak C, Payakkapol A (2016). Natural human *Plasmodium* infections in major *Anopheles* mosquitoes in western Thailand. Parasit Vectors..

[20] Ismail IAH, Notananda V, Schepens J. Studies on malaria and responses of *Anopheles balabacensis balabacensis* and *Anopheles minimus* to DDT residual spraying in Thailand. Geneva; World Health Organization; 1973. http://apps.who.int/iris/handle/10665/65676. Accessed 7 June 2019.

[21] Prajakwong S, Suwonkerd W, Chawprom S, Banchong-Aksorn T, Tsuda Y, Takagi M (2002). A field evaluation study on the effects of residual spray of Bifenthrin and Deltamethrin on *Anopheles minimus* population in Mae Hong Son Province, northern Thailand. Japanese J Trop Med Hyg..

[22] Pimnon S, Bhumiratana A (2018). Adaptation of *Anopheles* vectors to anthropogenic malaria-associated rubber plantations and indoor residual spraying: establishing population dynamics and insecticide susceptibility. Can J Infect Dis Med Microbiol..

[23] Suwonkerd W, Tsuda Y, Overgaard HJ, Chawprom S, Tuno N, Prajakwong S (2004). Changes in malaria vector densities over a twenty-three year period in Mae Hong Son province, northern Thailand. Southeast Asian J Trop Med Public Health.

[24] Suwonkerd W, Ritthison W, Ngo CT, Tainchum K, Bangs MJ, Chareonviriyaphap T. Vector biology and malaria transmission. In: Manguin S, editors. *Anopheles* mosquitoes—new insights into malaria vectors, Chapt 10. 2013. http://www.intechopen.com/books/anopheles-mosquitoes-new-insights-into-malaria-vectors/advances-and-perspectives-in-the-study-of-the-malaria-mosquito-anopheles-funestus. Accessed 7 June 2019.

[25] StataCorp. Stata statistical software: release 14. 2015.

[26] MEASURE Evaluation, MEASURE DHS, President’s Malaria Initiative, Roll Back Malaria Partnership, UNICEF, World Health Organization. Household survey indicators for malaria control. 2013. https://data.unicef.org/resources/household-survey-indicators-for-malaria-control-2013-edition/. Accessed 7 June 2019.

[27] QGIS Development Team. QGIS Geographic Information System. Open source geospatial foundation project. 2015. http://www.qgis.org/es/site/. Access 7 June 2019.

[28] Rattanarithikul R, Harbach RE, Harrison BA, Panthusiri P, Coleman RE, Richardson JH (2010). Illustrated keys to the mosquitoes of Thailand. VI. Tribe Aedini. SE Asian J Trop Med Public Health..

[29] Wirtz RA, Sattabongkot J, Hall TED, Burkot TR, Rosenberg R (1992). Development and evaluation of an enzyme-linked immunosorbent assay for *Plasmodium vivax*-VK247 sporozoites. J Med Entomol.

[30] Wirtz R, Avery M, Benedict M, Sutcliffe A. Plasmodium sporozoite ELISA. In: Methods in *Anopheles* research, 2nd ed. MR4, CDC. 2010.

[31] Seyoum A, Sikaala CH, Chanda J, Chinula D, Ntamatungiro AJ, Hawela M (2012). Human exposure to anopheline mosquitoes occurs primarily indoors, even for users of insecticide-treated nets in Luangwa Valley, South-east Zambia. Parasit Vectors.

[32] Guerra CA, Snow RW, Hay SI (2006). A global assessment of closed forests, deforestation and malaria risk. Ann Trop Med Parasitol.

[33] Tangena JAA, Thammavong P, Wilson AL, Brey PT, Lindsay SW (2016). Risk and control of mosquito-borne diseases in Southeast Asian rubber plantations. Trends Parasitol..

[34] Chang MS, Hii J, Buttner P, Mansoor F (1997). Changes in abundance and behaviour of vector mosquitoes induced by land use during the development of an oil palm plantation in Sarawak. Trans R Soc Trop Med Hyg.

[35] Abe T, Honda S, Nakazawa S, Tuong TD, Thieu NQ, Hung LX (2009). Risk factors for malaria infection among ethnic minorities in Binh Phuoc, Vietnam. SE Asian J Trop Med Public Health..

[36] Hiscox A, Khammanithong P, Kaul S, Sananikhom P, Luthi R, Hill N (2013). Risk factors for mosquito house entry in the Lao PDR. PLoS ONE.

[37] Tusting LS, Bottomley C, Gibson H, Kleinschmidt I, Tatem AJ, Lindsay SW (2017). Housing improvements and malaria risk in sub-Saharan Africa: a multi-country analysis of survey data. PLoS Med..

[38] Zanzibar Malaria Control Programme. Malaria elimination in Zanzibar. A feasibility assessment. 2009.

[39] Yukich J, Chitnis N. When can malaria control and elimination programs safely reduce vector control efforts? A simulation study. 2015. https://www.who.int/malaria/mpac/scaleback-vector-control-simulation-study.pdf. Accessed 7 June 2019.

[40] Cooke MK, Kahindi SC, Oriango RM, Owaga C, Ayoma E, Mabuka D (2015). “A bite before bed”: exposure to malaria vectors outside the times of net use in the highlands of western Kenya. Malar J..

[41] Atkinson JA, Fitzgerald L, Toaliu H, Taleo G, Tynan A, Whittaker M (2010). Community participation for malaria elimination in Tafea Province, Vanuatu: Part I. Maintaining motivation for prevention practices in the context of disappearing disease. Malar J..

[42] Bauch JA, Gu JJ, Msellem M, Mårtensson A, Ali AS, Gosling R (2013). Perception of malaria risk in a setting of reduced malaria transmission: a qualitative study in Zanzibar. Malar J.

[43] Zhu L, Müller GC, Marshall JM, Arheart KL, Qualls WA, Hlaing WM (2017). Is outdoor vector control needed for malaria elimination? An individual-based modelling study. Malar J.

[44] Cheng FY (1968). Responses of *Anopheles balabacensis* to various patterns of DDT-spraying of shelters in Sabah, East Malaysia. Bull World Health Organ..

[45] Adongo PB, Kirkwood B, Kendall C (2005). How local community knowledge about malaria affects insecticide-treated net use in northern Ghana. Trop Med Int Health..

[46] Yohannes K, Dulhunty JM, Kourleoutov C, Manuopangai VT, Polyn MK, Parks WJ (2000). Malaria control in central Malaita, Solomon Islands: 1. The use of insecticide-impregnated bed nets. Acta Trop.

[47] Stewart T, Marchand R. Factors that affect the success and failure of insecticide treated net programs for malaria control in SE Asia and the Western Pacific. 2001. https://www.who.int/malaria/publications/atoz/itn_r62.pdf. Accessed 7 June 2019.

[48] Nonaka D, Pongvongsa T, Nishimoto F, Nansounthavong P, Sato Y, Jiang H (2015). Households with insufficient bednets in a village with sufficient bednets: evaluation of household bednet coverage using bednet distribution index in Xepon District, Lao PDR. Trop Med Health..

[49] Van Roey K, Sovannaroth S, Sochantha T, Touch MS, Pigeon O, Sluydts V (2014). A phase III trial to evaluate the efficacy, fabric integrity and community acceptance of Netprotect^®^ using a recommended long-lasting insecticidal net as positive control. Malar J..

[50] National Centre for Parasitology, Entomology and Malaria Control; Malaria Consortium, UN Office for Project Services. Cambodia Malaria Survey 2013. 2013. https://www.malariaconsortium.org/media-downloads/624/Cambodia%20Malaria%20Survey%202013. Accessed 7 June 2019.

[51] National Malaria Control Programme Myanmar. Myanmar malaria indicator survey. Malaria Consortium, 2015.

[52] Grietens KP, Xuan XN, Ribera J, Duc TN, van Bortel W, Ba NT (2012). Social determinants of long lasting insecticidal hammock use among the Ra-glai ethnic minority in Vietnam: implications for forest malaria control. PLoS ONE.

[53] Gryseels C, Uk S, Sluydts V, Durnez L, Phoeuk P, Suon S (2015). Factors influencing the use of topical repellents: implications for the effectiveness of malaria elimination strategies. Sci Rep..

[54] Lindsay SW, Ewald JA, Samung Y, Apiwathnasorn C, Nosten F (1998). Thanaka (*Limonia acidissima*) and deet (di-methyl benzamide) mixture as a mosquito repellent for use by Karen women. Med Vet Entomol.

[55] McGready R, Simpson JA, Htway M, White NJ, Nosten F, Lindsay SW (2001). A double-blind randomized therapeutic trial of insect repellents for the prevention of malaria in pregnancy. Trans R Soc Trop Med Hyg.

[56] Trung HD, Van Bortel W, Sochantha T, Keokenchanh K, Briët OJT, Coosemans M (2005). Behavioural heterogeneity of *Anopheles* species in ecologically different localities in Southeast Asia: a challenge for vector control. Trop Med Int Health..

[57] Trung HD, Van Bortel W, Sochantha T, Keokenchanh K, Quang NT, Cong LD (2004). Malaria transmission and major malaria vectors in different geographical areas of Southeast Asia. Trop Med Int Heal..

[58] St. Laurent B, Oy K, Miller B, Gasteiger EB, Lee E, Sovannaroth S (2016). Cow-baited tents are highly effective in sampling diverse *Anopheles* malaria vectors in Cambodia. Malar J..

[59] Pooda HS, Rayaisse J-B, de Sale Hien DF, Lefèvre T, Yerbanga SR, Bengaly Z (2015). Administration of ivermectin to peridomestic cattle: a promising approach to target the residual transmission of human malaria. Malar J..

[60] Poché RM, Burruss D, Polyakova L, Poché DM, Garlapati RB (2015). Treatment of livestock with systemic insecticides for control of *Anopheles arabiensis* in western Kenya. Malar J..

[61] Lwetoijera D, Harris C, Kiware S, Dongus S, Devine GJ, McCall PJ (2014). Effective autodissemination of pyriproxyfen to breeding sites by the exophilic malaria vector *Anopheles arabiensis* in semi-field settings in Tanzania. Malar J..

[62] Fowkes FJI, Boeuf P, Beeson JG (2016). Immunity to malaria in an era of declining malaria transmission. Parasitology.

[63] Aung T, Lwin MM, Sudhinaraset M, Wei C (2016). Rural and urban disparities in health-seeking for fever in Myanmar: findings from a probability-based household survey. Malar J..

[64] Xu J-W, Xu Q-Z, Liu H, Zeng Y-R (2012). Malaria treatment-seeking behaviour and related factors of Wa ethnic minority in Myanmar: a cross-sectional study. Malar J..

[65] Sonkong K, Chaiklieng S, Neave P, Suggaravetsiri P (2015). Factors affecting delay in seeking treatment among malaria patients along Thailand–Myanmar border in Tak Province, Thailand. Malar J..

[66] Nguyen TN, von Seidlein L, Nguyen TV, Truong PN, Do Hung S, Pham HT (2018). The persistence and oscillations of submicroscopic *Plasmodium falciparum* and *Plasmodium vivax* infections over time in Vietnam: an open cohort study. Lancet Infect Dis..

[67] Tripura R, Peto TJ, Chalk J, Lee SJ, Sirithiranont P, Nguon C (2016). Persistent *Plasmodium falciparum* and *Plasmodium vivax* infections in a western Cambodian population: implications for prevention, treatment and elimination strategies. Malar J..

[68] Betuela I, Rosanas-Urgell A, Kiniboro B, Stanisic DI, Samol L, de Lazzari E (2012). Relapses contribute significantly to the risk of *Plasmodium vivax* infection and disease in Papua New Guinean children 1–5 years of age. J Infect Dis.

[69] Ross A, Koepfli C, Schoepflin S, Timinao L, Siba P, Smith T (2016). The incidence and differential seasonal patterns of *Plasmodium vivax* primary infections and relapses in a cohort of children in Papua New Guinea. PLoS Negl Trop Dis..

[70] WHO Global Malaria Programme. Guidance for countries on combining indoor residual spraying and long-lasting insecticidal nets. Geneva: World Health Organization; 2014. https://www.who.int/malaria/publications/atoz/who-guidance-combining-irs_llins-mar2014.pdf. Accessed 7 June 2016.

[71] Chareonviriyaphap T, Bangs MJ, Suwonkerd W, Kongmee M, Corbel V, Ngoen-Klan R (2013). Review of insecticide resistance and behavioral avoidance of vectors of human diseases in Thailand. Parasit Vectors.

[72] Overgaard HJ, Sandve SR, Suwonkerd W (2005). Evidence of anopheline mosquito resistance to agrochemicals in northern Thailand. Southeast Asian J Trop Med Public Health.

[73] Chaumeau V, Cerqueira D, Zadrozny J, Kittiphanakun P, Andolina C, Chareonviriyaphap T (2017). Insecticide resistance in malaria vectors along the Thailand–Myanmar border. Parasit Vectors..

[74] Sumarnrote A, Overgaard HJ, Marasri N, Fustec B, Thanispong K, Chareonviriyaphap T (2017). Status of insecticide resistance in *Anopheles* mosquitoes in Ubon Ratchathani province, Northeastern Thailand. Malar J..

[75] Limsakul A, Singhruck P (2016). Long-term trends and variability of total and extreme precipitation in Thailand. Atmos Res.

[76] Global Solutions Group, World Bank Group. DRM & Reslience updates. El Niño Briefing note: Potential impacts and options for WBG response. East Asia & Pacific (EAP) Regional perspective. 2016. https://siteresources.worldbank.org/INTSOUTHASIA/Resources/223497-1378327471830/9316011-1460017133668/ElNinoBRIEFINGNOTEGlobal_EAP_Perspective_March_28_clean.pdf. Accessed 7 June 2019.

[77] Sungvornyothin S, Muenvorn V, Garros C, Manguin S, Prabaripai A, Bangs MJ (2006). Trophic behavior and biting activity of the two sibling species of the *Anopheles minimus* complex in western Thailand. J Vector Ecol.

[78] Baimai V, Kijchalao U, Sawadwongporn P, Green CA (1988). Geographic distribution and biting behaviour of four species of the *Anopheles dirus* complex (Diptera: Culicidae) in Thailand. Southeast Asian J Trop Med Public Health.

[79] Bhumiratana A, Intarapuk A, Sorosjinda-Nunthawarasilp P, Maneekan P, Koyadun S (2013). Border malaria associated with multidrug resistance on Thailand–Myanmar and Thailand–Cambodia borders: transmission dynamic, vulnerability, and surveillance. Biomed Res Int.

