# Correction to: Transmission risk beyond the village: entomological and human factors contributing to residual malaria transmission in an area approaching malaria elimination on the Thailand–Myanmar border

**DOI:** 10.1186/s12936-019-2881-0

**Published:** 2019-07-24

**Authors:** Hannah M. Edwards, Patchara Sriwichai, Kirakorn Kirabittir, Jetsumon Sattabongkot, Irwin F. Chavez, Jeffrey Hii

**Affiliations:** 1 Department of Disease Epidemiology, Imperial College London, London, UK; 2 Malaria Consortium Asia, Faculty of Tropical Medicine, Mahidol University, 420/6 Rajvithi Road, Bangkok, 10400 Thailand; 3 Department of Entomology, Faculty of Tropical Medicine, Mahidol University, 420/6 Rajvithi Road, Bangkok, 10400 Thailand; 4 Mahidol Vivax Research Unit, Faculty of Tropical Medicine, Mahidol University, 420/6 Rajvithi Road, Bangkok, 10400 Thailand; 5 Department of Tropical Hygiene, Faculty of Tropical Medicine, Mahidol University, 420/6 Rajvithi Road, Bangkok, 10400 Thailand

## Correction to: Malar J (2019) 18:221 10.1186/s12936-019-2852-5

Following publication of the original article [1], the authors advised of two errors present in the article: one concerning two author names and the other missing funding details.

The errors are:

1. The named authors of ‘Jetsumon Prachumsri’ and ‘Kirakorn Kirabittir’ are incorrect and should read respectively as:
  ‘Jetsumon Sattabongkot’ and ‘Kirakorn Kiattibutr’

2. Under the ‘Study area’ sub-section of the ‘Methods’ section, this sentence is missing funding details:

- “As part of a concurrent longitudinal study in the area, prevalence by microscopy in the three villages was found to be 0.71%, 0.89% and 0.27% in January, May and November 2016, respectively (Fig. 1); all infections were caused by *Plasmodium vivax*.”

However, the sentence should read as:

- “As part of a concurrent longitudinal study in the area from Mahidol University (ICEMR and D43 funded Projects U19AI0819672 and D43TW006571), prevalence by microscopy in the three villages was found to be 0.71%, 0.89% and 0.27% in January, May and November 2016, respectively (Fig. 1); all infections were caused by *Plasmodium vivax*.”

The authors apologize for these errors.
