# Supplementary material for: Transmission risk beyond the village: entomological and human factors contributing to residual malaria transmission in an area approaching malaria elimination on the Thailand–Myanmar border
Source: Malar J. 2019 Jul 1;18:221. doi: 10.1186/s12936-019-2852-5 (PMC6604376; doi:10.1186/s12936-019-2852-5)
Supplement: Supplementary file 1 — Additional file 1: Table S1. Demographics of the surveyed adult (aged 18 years and over) population in the three study villages of Tha Song Yang district. [file 12936_2019_2852_MOESM1_ESM.pdf]

Table S1. Demographics of the surveyed adult (aged 18 years and over) population in the three study villages of Tha Song Yang district

	Komonae (n = 115)			Suan Oi (n = 265)			Pha Man (n = 50)			Total (n = 430)		
	n	%	95% CI	n	%	95% CI	n	%	95% CI	n	%	95% CI
**Sex**												
Male	51	44.35	35.49-53.58	113	42.64	36.79-48.70	22	44	30.86-58.04	186	43.26	38.63-48.00
Female	64	55.65	46.42-64.51	152	57.36	51.30-63.21	28	56	41.96-69.14	244	56.74	52.00-61.37
**Nationality**												
Thai	22	19.13	12.90-27.42	80	30.42	25.14-36.27	12	24	14.06-37.87	114	26.64	22.65-31.04
MM	93	80.87	72.58-87.10	183	69.58	63.73-74.86	38	76	62.13-85.94	314	73.36	68.96-77.35
**Ethnic group**												
Thai	3	2.61	0.84-7.84	17	6.42	4.02-10.10	1	2	0.27-13.17	21	4.88	3.20-7.39
Karen	112	97.39	92.16-99.16	248	93.58	89.90-95.98	49	98	86.83-99.73	409	95.12	92.61-96.80
**Residency**												
Permanent	114	100		259	98.48	96.00-99.43	50	100		423	99.06	97.52-99.65
Temp. >6m	**0**			3	1.14	0.37-3.49	0			3	0.7	0.23-2.16
Temp <6m	**0**			1	0.38	0.05-2.67	0			1	0.23	0.03-1.66
**Occupation**												
Farmer	62	53.91	44.71-62.86	51	19.25	14.92-24.47	22	44	30.86-58.04	135	31.4	27.17-35.96
Labourer	23	20	13.63-28.37	106	40	34.25-46.04	13	26	15.63-39.99	142	33.02	28.72-37.63
Sales	2	1.74	0.43-6.75	26	9.81	6.75-14.04	2	4	0.98-14.87	30	6.98	4.91-9.82
Forestry	0			1	0.38	0.05-2.65	1	2	0.27-13.17	2	0.47	0.12-1.85
Cattle trader	0			2	0.75	0.19-2.98	0			2	0.47	0.12-1.85
Unemployed	2	1.74	0.43-6.75	18	6.79	4.31-10.54	4	8	3.00-19.67	24	5.58	3.76-8.20
Other	4	4.3	1.61-10.99	17	7.87	4.93-12.33	0			21	5.98	3.92-9.02
Housewife	22	19.13	12.90-27.42	49	18.49	14.25-23.65	8	16	8.14-29.06	79	18.37	14.98-22.33
