# Supplementary material for: Transmission risk beyond the village: entomological and human factors contributing to residual malaria transmission in an area approaching malaria elimination on the Thailand–Myanmar border
Source: Malar J. 2019 Jul 1;18:221. doi: 10.1186/s12936-019-2852-5 (PMC6604376; doi:10.1186/s12936-019-2852-5)
Supplement: Supplementary file 2 — Additional file 2: Table S2. Utilization of bed nets the previous night by demographic factor among the surveyed adult population. [file 12936_2019_2852_MOESM2_ESM.pdf]

Table S2. Utilisation of bed nets the previous night by demographic factor among the surveyed adult population

		Didn't sleep under net			Slept under conventional net			Slept under ITN/LLIN		
		**n**	**%**	**95% CI**	**n**	**%**	**95% CI**	**n**	**%**	**95% CI**
**Total**		28	6.5	4.5-9.3	60	14.0	11.0-17.6	342	79.5	75.4-83.1
**Village**	Komonae	13	11.3	6.7-18.6	3	2.6	0.8-7.8	99	86.1	78.4-91.3
	Suan Oi	14	5.3	3.2-8.7	48	18.1	13.9-23.2	203	2.0	71.1-81.3
	Pha Man	1	2.0	0.3-13.2	9	18.0	9.6-31.3	40	80.0	66.5-89.0
**Sex**	Male	18	9.7	6.2-14.9	23	12.4	8.3-18.0	145	78.0	71.4-83.4
	Female	10	4.1	2.2-7.5	37	15.2	11.2-20.3	197	80.7	75.3-85.2
**Nationality**	Thai	8	7.0	3.5-13.5	31	27.2	19.8-36.1	75	65.8	56.6-74.0
	MM	20	6.4	4.1-9.7	29	9.2	6.5-13.0	265	84.4	79.9-88.0
**Ethnic group**	Thai	1	4.8	0.6-28.3	3	14.3	4.5-36.9	17	81.0	58.1-92.9
	Karen	27	6.6	4.6-9.5	57	13.9	10.9-17.7	325	79.5	75.3-83.1
