# Supplementary material for: Transmission risk beyond the village: entomological and human factors contributing to residual malaria transmission in an area approaching malaria elimination on the Thailand–Myanmar border
Source: Malar J. 2019 Jul 1;18:221. doi: 10.1186/s12936-019-2852-5 (PMC6604376; doi:10.1186/s12936-019-2852-5)
Supplement: Supplementary file 3 — Additional file 3: Table S3. Calculation of indoor and outdoor exposure risk for users and non-users of LLINs in Pha Man. [file 12936_2019_2852_MOESM3_ESM.pdf]

Table S3. Calculation of indoor and outdoor exposure risk for users and non-users of LLINs in Pha Man.

* PERSONAL PROTECTION PROVIDED BY AN LLIN (Mean of Okumu et al 2011 Malaria Journal 10: 208) = 0.937

An. maculatus s.l.:

Hours	IHLC biting rate (bpn)	OHLC biting rate (bpn)	Proportion of the population indoors	Indoor exposure without use of ITN	Indoor exposure with use of ITN*	Outdoor exposure	Indoor exposure prevented by ITN
6pm-7pm	0.08	0.42	0.66	0.06	0.00	0.14	0.05
7pm-8pm	0.33	0.92	0.74	0.25	0.02	0.24	0.23
8pm-9pm	0.33	0.25	0.88	0.29	0.02	0.03	0.27
9pm-10pm	0.42	0.00	0.94	0.39	0.02	0.00	0.37
10pm-11pm	0.17	0.33	0.86	0.14	0.01	0.05	0.13
11pm-12pm	0.08	0.00	1.00	0.08	0.01	0.00	0.08
12pm-1am	0.00	0.08	1.00	0.00	0.00	0.00	0.00
1am-2am	0.08	0.00	1.00	0.08	0.01	0.00	0.08
2am-3am	0.00	0.08	1.00	0.00	0.00	0.00	0.00
3am-4am	0.00	0.00	1.00	0.00	0.00	0.00	0.00
4am-5am	0.00	0.08	1.00	0.00	0.00	0.00	0.00
5am-6am	0.17	0.42	0.97	0.16	0.01	0.01	0.15
ALL NIGHT TOTAL				**1.46**	**0.09**	**0.47**	**1.37**

Proportion of human exposure occurring indoors for non-users of LLINs (πi): 0.76

Proportion of human exposure occurring indoors for users of LLINs (πi,n): 0.16

An. minimus s.l.:

Hours	IHLC biting rate (bpn)	OHLC biting rate (bpn)	Proportion of the population indoors	Indoor exposure without use of ITN	Indoor exposure with use of ITN*	Outdoor exposure	Indoor exposure prevented by ITN
6pm-7pm	0.00	0.08	0.66	0.00	0.00	0.03	0.00
7pm-8pm	0.17	0.17	0.74	0.12	0.01	0.04	0.12
8pm-9pm	0.17	0.25	0.88	0.15	0.01	0.03	0.14
9pm-10pm	0.42	0.58	0.94	0.39	0.02	0.04	0.37
10pm-11pm	1.25	1.17	0.86	1.08	0.07	0.16	1.01
11pm-12pm	0.42	0.25	1.00	0.42	0.03	0.00	0.39
12pm-1am	0.25	0.42	1.00	0.25	0.02	0.00	0.23
1am-2am	1.17	0.42	1.00	1.17	0.07	0.00	1.09
2am-3am	0.58	0.08	1.00	0.58	0.04	0.00	0.55
3am-4am	0.42	0.08	1.00	0.42	0.03	0.00	0.39
4am-5am	0.33	0.17	1.00	0.33	0.02	0.00	0.31
5am-6am	0.50	0.58	0.97	0.49	0.03	0.02	0.45
ALL NIGHT TOTAL				**5.39**	**0.34**	**0.32**	**5.05**

Proportion of human exposure occurring indoors for non-users of LLINs (πi): 0.94

Proportion of human exposure occurring indoors for users of LLINs ($\pi i,n$): 0.52
